# The Structural and Functional Study of Efflux Pumps Belonging to the RND Transporters Family from Gram-Negative Bacteria

**DOI:** 10.3390/antibiotics11040429

**Published:** 2022-03-23

**Authors:** Attilio Vittorio Vargiu, Gilles Phan, Henrietta Venter, Isabelle Broutin

**Affiliations:** 1 Department of Physics, University of Cagliari, S.P. 8 km 0.700, 09042 Monserrato, Italy; vargiu@dsf.unica.it; 2 Laboratoire CiTCoM, Faculté de Santé, Université Paris Cité, CNRS, 75006 Paris, France; gilles.phan@parisdescartes.fr; 3 Health and Biomedical Innovation, Clinical and Health Sciences, University of South Australia, Adelaide, SA 5000, Australia; rietie.venter@unisa.edu.au

Antimicrobial-resistant bacterial infections are a major and costly public health concern. Several pathogens are already pan-resistant, representing a major cause of mortality in patients suffering from nosocomial infections. Drug efflux pumps, which remove compounds from the bacterial cell, thereby lowering the antimicrobial concentration to sub-toxic levels, play a major role in multidrug resistance (MDR).

Gram-negative bacteria are particularly resistant, and some are identified by the World Health Organization as the pathogens most urgently in need of new antimicrobial drug discovery. The most clinically relevant efflux pumps in Gram-negative bacteria belong to the Resistance Nodulation cell Division (RND) family, which form a tripartite macromolecular assembly spanning both membranes and the periplasmic space of Gram-negative organisms.

Along with functional studies and in silico approaches, many structures of the individual components, as well as the fully assembled pumps from several pathogens, have been solved. Nevertheless, many questions concerning the assembly and the mechanism of efflux remain, while there are still no efflux pump inhibitors available in clinical treatment.

This Special Issue is a representation of the current knowledge regarding the mechanism, regulation, dissemination, etc., of these efflux pumps.

Bernal et al. [1] explored the putative pathway used by charged substrates in the AcrAD-TolC RND efflux pump of *Salmonella typhimurium* (St) using site-directed mutational analysis, combined with complementation and minimum inhibition concentration (MIC) measurements, in an *E. coli* ∆*acrB*∆*acrD* strain. They highlighted several amino acids of the deep binding pocket, the access pocket, and the TM1/TM2 groove region as being involved in the specificity of AcrD efflux for aminoglycosides and dianionic β-lactams. They also confirmed that St-AcrD is able to function with *E. coli* AcrA and TolC, and reported temocillin, dicloxacillin, cefazolin and fusidic acid as substrates of AcrD, contrary to piperacillin, which is not a substrate. Their results underline the need to explore single mutation variants *in cellulo*, instead of a simple extrapolation of the effect based on a model.

As the efflux of drugs in Gram-negative bacteria requires the formation of a long tripartite assembly to pass through the two membranes, several groups have attempted to understand the assembly process.

Boyer et al. [2] investigated the dynamic and selectivity of the *Pseudomonas aeruginosa* MexAB-OprM efflux pump assembly. They studied the effect of the gain of function mutation Q93R, identified in the periplasmic adaptor protein (PAP) MexA, and its capacity to form a functional assembly with different outer membrane factors (OMF) (OprM, OprN, TolC, and OprM-ΔCter truncated of its 13 last amino acids of unresolved structure). Using several biophysical approaches (size-exclusion chromatography, biolayer interferometry, negative staining electron microscopy) combined with minimum inhibition concentration (MIC) measurements, they highlighted a modulation effect in the assembly process efficacy, involving molecular determinants other than the tip-to-tip OMF–PAP interface.

Rajapaksha et al. [3] studied the assembly process of the *E. coli* AcrAB-TolC efflux pump in cellulo. They studied the effect of the overexpression of the proteins forming the AcrAB-TolC efflux pump bearing “loss of function mutations”, using MIC measurements for different antibiotics in the context of gene-deleted and wild-type *E. coli* strains. They observed no significant drop in the efflux activity, indicating that the RND pump assembly process in Gram-negative bacteria is a precisely controlled process that prevents the formation of functionless complexes. In addition, they performed competition experiments, which highlighted that the dissociation kinetics of the AcrAB complex are very slow. Together, these results indicate that the assembly of the AcrAB-TolC complex has a proof-read mechanism that effectively eliminates the formation of a futile pump complex.

Webber et al. [4] investigated the AcrAB-TolC efflux pump opening mechanism. They tried to explain how the drug transport induces assembly and opening of the AcrA and TolC partners. They compared the resting and transport states of the AcrAB-TolC pump, for which several structures exist in the presence and absence of a substrate. They indicate that the “assembled opened” conformation state has lower energy than the resting state, suggesting the need for energy input to perform the conformation changes that are required to go back to the resting state after the efflux of a substrate. From this observation, and by using distance matrices supplemented with evolutionary coupling data and buried surface area measurements, they propose a new allosteric model for the function of the pump.

AcrAB-TolC and MexAB-OprM are the two most-studied efflux pumps, but important information can also be acquired from the characterization of RND efflux pumps by looking at emerging pathogens and their role in antimicrobial resistance.

Mateus et al. [5] characterized three efflux systems found in an emergent enteropathogen, *Aliarcobacter butzleri*, looking at their involvement in resistance and virulence. They identified the substrates of AreABC, AreDEF and AreGHI by performing MIC measurements and ethidium bromide accumulation experiments, and analyzed their implication in the resistance to oxidative and bile stress, in bacterial fitness, their impact on motility and biofilm formation ability, and their ability to survive in human serum and adhere to intestinal cells. They show that these three RND pumps could be considered putative targets for new therapeutic strategies to fight infections with this emerging pathogen.

In contrast with most pathogens, only AcrB has been studied for its impact on resistance in *E. coli*. Schuster et al. [6] studied the contribution of the MdtF pump, functioning with MdtE and TolC, to drug resistance in an MDR *E. coli* isolated from a patient. By comparing the MIC values of different drugs and the accumulation and the efflux of different dyes in the original strain and in the deleted strain of the genes coding for AcrB, TolC, or both AcrB and MdtF, they showed a limited contribution of MdtF to the antibiotic resistance profile of this MDR *E. coli* isolate, but a remarkable capacity to export dyes.

Finally, as MDR bacteria are spreading wordlwide, it is most important to evaluate their diversity and predominance in clinics.

Scoffone et al. [7] listed the RND efflux pumps of the different Gram-negative bacterial species found in the lung of 70,000 patients worldwide suffering from cystic fibrosis (CF). They focused their review on the four species that are predominantly encountered (*Pseudomonas aeruginosa*, *Burkholderia cenocepacia*, *Achromobacter xylosoxidans*, *Stenotrophomonas maltophilia*). For each species, they formed the link between the CF lung environment modifications that can append during the patients’ life and the over-expression of specific RND pumps that participate in the bacteria adaptation. They also provide an overview of the efflux pumps inhibitors that are described as efficient for these pumps.

Zwama and Nishiro [8] performed a genetic evolution analysis on 135 RND efflux pumps to highlight the conserved and variable domains in the RNDs’ structure. They also made an inventory of all the mutants that have been described in RND transporters from different clinically, environmentally and laboratory-evolved Gram-negative bacterial strains (*Escherichia coli*, *Salmonella enterica*, *Neisseria gonorrhoeae*, *Pseudomonas aeruginosa*, *Acinetobacter baumannii* and *Legionella pneumophila*), which displayed increased antimicrobial resistance. They show that the TM domain, bearing the motor, is largely conserved, as well as three residues located in the loops linking this domain to the binding domain. The binding domain presents more versatility. However, some strategic amino acid positions are highlighted as possible clues to the selectivity for certain antibiotic families. In conclusion, they underlined the worrying resistance adaptation of several bacterial strains by acquiring mutations directly in the RND-coding genes.

Davin-Regli et al. [9] provided an overview of the prevalence of the main efflux pumps observed in clinical practice and listed the clinical impact situation for some of the most concerning Gram-negative species. They highlighted the need to develop new efflux pump inhibitors, and provided an overview of the different methods used to measure the inhibition power of tested molecules.

This collection of data could serve as a basis for antimicrobial drug discovery that aims to inhibit drug efflux pumps to reverse resistance in some of the most resistant pathogens.
